# Prolonged migraine aura resembling ischemic stroke following CoronaVac vaccination: an extended case series

**DOI:** 10.1186/s10194-022-01385-0

**Published:** 2022-01-21

**Authors:** Nijasri C. Suwanwela, Naruchorn Kijpaisalratana, Supatporn Tepmongkol, Wanakorn Rattanawong, Pongpat Vorasayan, Chutibhorn Charnnarong, Jarturon Tantivattana, Sirigunya Roongruang, Tatchaporn Ongphichetmetha, Poonnakarn Panjasriprakarn, Aurauma Chutinet, Wasan Akarathanawat, Jeffrey L. Saver

**Affiliations:** 1 Division of Neurology, Department of Medicine, Faculty of Medicine, Chulalongkorn University, Rama IV Road, Bangkok, 10330 Thailand; 2 Chula Neuroscience Center, King Chulalongkorn Memorial Hospital, Bangkok, Thailand; 3 Chulalongkorn Stroke Center, King Chulalongkorn Memorial Hospital, Bangkok, Thailand; 4 Division of Academic Affairs, Faculty of Medicine, Chulalongkorn University, Bangkok, Thailand; 5 Division of Nuclear Medicine, Department of Radiology, Faculty of Medicine, Chulalongkorn University, Bangkok, Thailand; 6 Chulalongkorn University Biomedical Imaging Group (CUBIG), Department of Radiology, Faculty of Medicine, Chulalongkorn University, Bangkok, Thailand; 7 Department of Medicine, Faculty of Medicine, King Mongkut’s Institute of Technology Ladkrabang, Bangkok, Thailand; 8 Faculty of Medicine, Chulalongkorn University, Bangkok, Thailand; 9 Department of Radiology, Faculty of Medicine, Chulalongkorn University, Bangkok, Thailand; 10 Golden Jubilee Medical Center, Faculty of Medicine, Siriraj Hospital, Mahidol University, Bangkok, Thailand; 11 Department of Neurology, David Geffen School of Medicine at UCLA, Los Angeles, CA USA

**Keywords:** CoronaVac vaccine, Cortical spreading depression, Sinovac, Neurological deficit, COVID-19

## Abstract

**Background:**

After the initiation of the COVID-19 vaccination program in Thailand, thousands of patients have experienced unusual focal neurological symptoms. We report 8 patients with focal neurological symptoms after receiving inactivated virus vaccine, CoronaVac.

**Case series:**

Patients were aged 24–48 years and 75% were female. Acute onset of focal neurological symptoms occurred within the first 24 h after vaccination in 75% and between 1-7d in 25%. All presented with lateralized sensory deficits, motor deficits, or both, of 2–14 day duration. Migraine headache occurred in half of the patients. Magnetic resonance imaging of the brain during and after the attacks did not demonstrate any abnormalities suggesting ischemic stroke. All patients showed moderately large regions of hypoperfusion and concurrent smaller regions of hyperperfusion on SPECT imaging while symptomatic. None developed permanent deficits or structural brain injury.

**Discussions:**

Here, we present a case series of transient focal neurological syndrome following Coronavac vaccination. The characteristic sensory symptoms, history of migraine, female predominant, and abnormal functional brain imaging without structural changes suggest migraine aura as pathophysiology. We propose that pain related to vaccine injection, component of vaccine, such as aluminum, or inflammation related to vaccination might trigger migraine aura in susceptible patients.

**Supplementary Information:**

The online version contains supplementary material available at 10.1186/s10194-022-01385-0.

## Background

Neurologic complications of COVID-19 vaccination are rare, but reported definite and possible nervous system adverse events have included cerebral venous thrombosis, Guillain-Barre syndrome, postural orthostatic tachycardia, and immunization stress-related response. The COVID-19 vaccination program in Thailand started in February 2021, with the two available vaccines at that time being CoronaVac (Sinovac Biotech, China) and ChAdOx1 (AstraZeneca/Oxford, UK). Both were approved by the Thai Food and Drug Administration. However, after mass vaccination, cases with a distinctive, novel focal neurological syndrome have begun to emerge nationwide among those receiving CoronaVac.

The very first case report regarding this phenomenon was published in this journal [1]. After thousands of injections in our hospital, several more cases have been consulted by the neurology team for further evaluation as acute stroke was suspected. Interestingly, these unusual presentations and findings were consistent among the patients. Here we present eight cases of this immunization-related focal neurological syndrome that underwent extensive investigation in our hospital. This work was presented as an abstract at the World Congress of Neurology (WCN 2021) in Rome, Italy (3–7 October 2021) [2].

## Case series

### Case ascertainment

As of May 9, 2021, at King Chulalongkorn Memorial Hospital, 13,194 individuals had been administered first doses of the CoronaVac vaccine, and among them, 4064 s doses were administered. There were 2553 individuals to whom first doses of the ChAdOx1 vaccine were administered. None received a second dose of the ChAdOx1 vaccine. According to our post-vaccination surveillance record, 49 of the total 15.747 patients (0.31%) were reported to have focal neurological symptoms. All received the CoronaVac vaccine. Among these 49 patients, unilateral sensory disturbance was the most frequent focal symptom; hemiparesis occurred less often but was more disabling. Headaches were sometimes, but not always, part of the clinical picture as well. The onset of focal neurological symptoms was within 24 h of vaccination in a majority of patients and within 7 days in all. Symptom duration most commonly lasted for 2–4 days, and in all cases, symptoms resolved within 2 weeks. Of the 49 cases, many did not seek medical attention due to the mild sensory symptoms.

In 8 of the cases, the severity of neurologic symptoms was sufficient to prompt a detailed neurodiagnostic investigation, with the findings delineated below.

### Clinical presentation

Individual characteristics and clinical courses of the 8 patients are shown in Table 1**,** and Supplement Results 1 provides detailed case descriptions for each patient. Overall, patients included 6 women and 2 men, with a mean age of 32 years and an age range of 24–48 years. Acute onset of focal neurological symptoms transpired within the first 2 h after vaccination in 38% of patients, between 2 and 24 h in 38%, and between 1 and 7 days in 25%. The focal neurological symptoms occurred ipsilateral to the injection site in 5 patients and contralateral in 3. All patients received the CoronaVac vaccine. The neurological symptoms developed after the first dose in 7 patients and after the second dose in 1 patient. The past medical history included migraine in 4 (1 with aura) of 6 patients with documented headache history and autoimmune disorders in 2 patients (Graves’ disease and systemic lupus erythematosus). Half of the patients, including the two men, had a body mass index (BMI) of over 30.

**Table 1:** Characteristics of the Patients

Characteristics	Patient 1	Patient 2	Patient 3	Patient 4	Patient 5	Patient 6	Patient 7	Patient 8
Age (year)	24	24	42	48	47	29	29	40
Sex	Female	Male	Female	Female	Male	Female	Female	Female
Past medical history	–	–	–	–	Hypertension	Graves’ Disease	endometriosis	SLE
History of migraine Current medication (s)	Yes	Yes	Yes	–	-candesartan, atenolol	-methimazole	-contraceptive pills	Yes hydroxychloroquine
BMI	32.8	37.18	39.04	24.03	37.58	19.14	20.02	18.9
Symptom onset after vaccination	20 min	1 day	7 h	1 day	2 h	7 days	7 days	6 h
Side of injection	Left	Left	Left	Left	Left	Left	Left	Left
Type of vaccine	CoronaVac	CoronaVac	CoronaVac	CoronaVac	CoronaVac	CoronaVac	CoronaVac	CoronaVac
Side of neuro deficit	Left	Left	Right	Left	Right	Right	Left	Left
Symptoms & signs								
Sensory: location	Tingling progress to numbness at Lt. arm, leg	Numbness Lt. side of face, neck, arm	–	Numbness Lt. Side of face, neck, arm	Tingling progress to numbness at Rt. fingertips, hand, perioral, foot	Rt. side of face, perioral and Rt. leg numbness	Tingling and numbness at Lt. face, perioral, arm, leg	Tingling at Lt. arm progress to numbness perioral, Lt. cheek
Sensory: progression	15 mins	Within 1 h	–	Not known	6 h	Few mins	10 mins	1 h
Motor	Lt. hemiparesis grade 3	Lt hemiparesis grade 4	Rt. Hemiparesis grade 4	Lt. arm monoparesis grade 4	–	Asymmetric nasolabial folds	–	–
Headache	Lt. temporal pulsatile headache 1 day later	Lt. temporal & occipital headache 1 day later	Rt. temporal & periorbital headache 1 day later	Occipital headache on the same day	–	–	–	–
Nausea/vomiting	Yes	Yes	–	–	–	–	–	–
Dizziness/vertigo	Yes	–	–	–	–	–	–	–
Visual phenomena	flashing light	blurred vision	–	–	–	–	–	–
Symptom duration (days)	10	2	3	14	4	4	5	12
MRI	no infarction	no infarction	no infarction	no infarction	no infarction	no infarction	no infarction	no infarction
MRA	mild irregularity	no abnormalities	no abnormalities	no abnormalities	no abnormalities	no abnormalities	no abnormalities	no abnormalities

Overall, neurologic symptoms fell into three major categories: sensory symptoms, motor symptoms, and headache. Unilateral sensory disturbance was the most common symptom, present in seven out of eight patients. The distribution of sensory disturbance was unilateral in all 7 cases, typically involving the face with predilection for the perioral area, hand, and arm. In five cases, patients described an initial tingling sensation followed by numbness. In all, the sensory symptoms started in the hand and progressed to the ipsilateral arm, face, and/or leg. The pace of the march of sensory symptoms was over a few minutes to 20 min in 3 cases. Two patients were not certain about the pace of the sensory march but stated that the numbness was progressive and reached its maximum within 1 h. One patient stated that the sensory symptoms progressed over six hours. In one patient, the numbness was accidentally noticed while she was applying a cream to her arm and chest. On physical examination, all patients with sensory symptoms reported decreased pinprick sensation and light touch on the same side as the sensory symptoms. Hemisensory loss involving the face, arm, and leg was found in 6 patients. The distribution of sensory loss was not at the midline, and there was no splitting of vibration sense when a tuning fork was placed on the frontal bone in all patients.

Motor symptoms were the second most common clinical presentation, occurring in four patients. The symptoms ranged from subtle facial weakness to a moderate degree of hemiparesis (3–4 out of 5 on the Medical Research Council muscle strength scale). On physical examination, objective facial weakness was observed in 2 patients. In all patients, the distribution of weakness was compatible with a pyramidal pattern. Hyperreflexia on the affected side was observed in 2 patients. There was no evidence of functional weakness on Hoover’s test in the 2 patients who reported leg weakness. In one patient, Hoover’s test was equivocal, but unilateral hyperreflexia was demonstrated. The motor weakness was usually distributed within the same region as the sensory symptoms.

Headaches were reported in four patients (50%). Three of them were unilateral with throbbing characteristics and occurred 1 day after the presenting symptoms. Two were accompanied by visual phenomena (1 flashing lights, 1 blurred vision). The duration of the headache in all of the patients met the duration of migraine headaches.

Symptom duration was 2–4 days in 75% of patients, and in all cases, symptoms resolved within 2 weeks.

### Neuroimaging findings

All patients underwent magnetic resonance imaging (MRI) and gadolinium contrast-enhanced magnetic resonance angiography (MRA) of the brain while still having neurological deficits. All MRIs were performed using a 3-Tesla MR scanner (Skyra, Siemens, Erlangen, Germany). MRIs, including diffusion-weighted images, were normal in all cases. MRAs showed no significant arterial stenosis. In one case (Case 1), only mild irregularities of the pericallosal branch of the intracranial artery were reported in one case (Case 1).

**Fig. 1:**
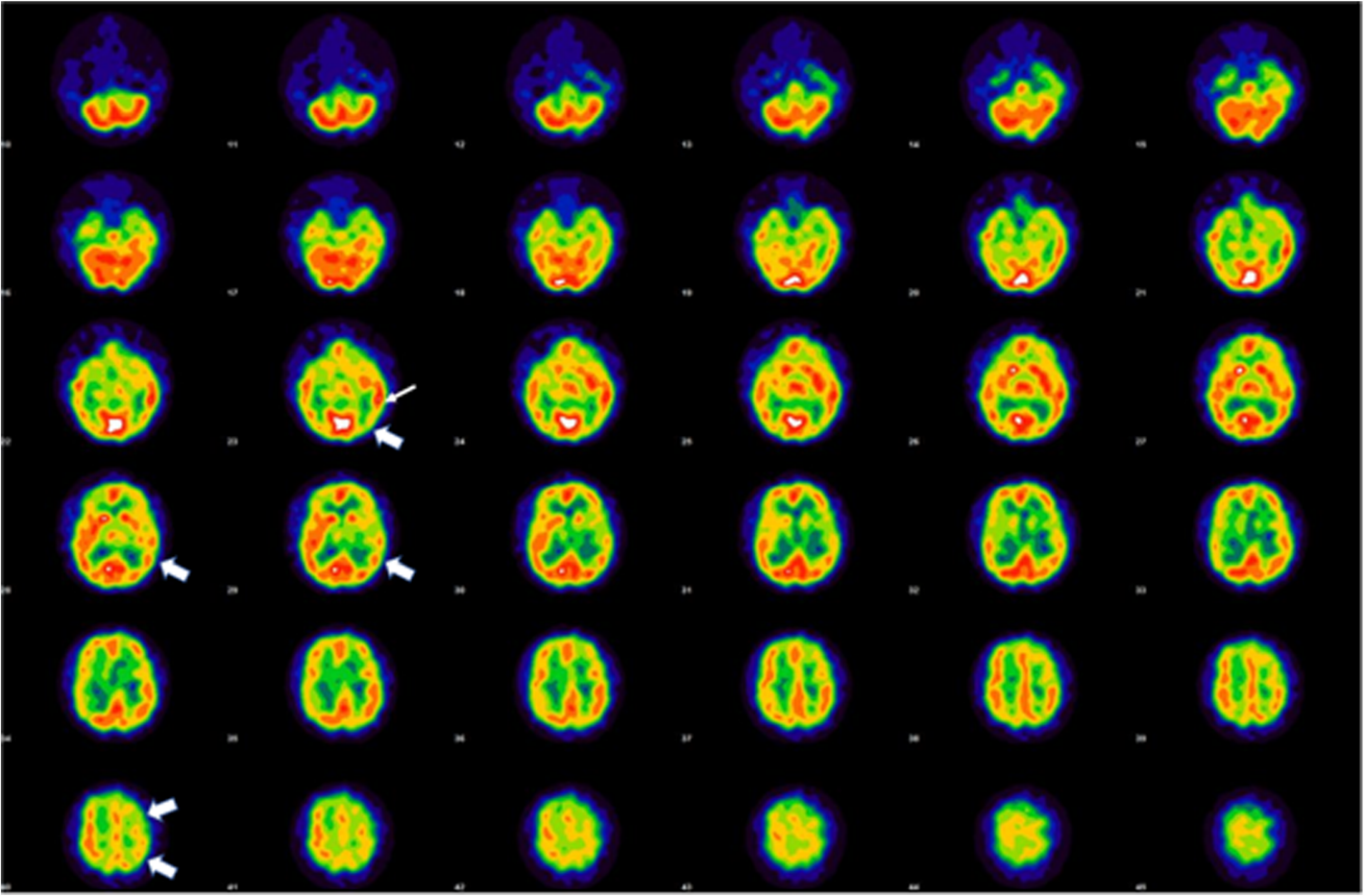
Axial single photon emission computer tomography with Tc-99 m ECD shows hypoperfusion to the left parietal area (Bold arrow) with adjacent small area of hyperperfusion in the left frontotemporal area (Thin arrow)

Single-photon emission computed tomography (SPECT) was performed by intravenous injection of Tc-99 m ethylcysteinate dimer (ECD) at 11.1 MBq/kg (0.3 mCi/kg). All patients had SPECT studies while having the focal neurological symptom. Patients were placed in a dimly lit room with low noise, and were instructed to keep their eyes open, have no interactions with other people, and stay comfortably still for 30 min. Acquisition started 30 min after injection using a dual-headed GE Discovery 670 (Chicago, USA). The SPECT/CT machines were equipped with low-energy, high-resolution, parallel hole collimators. The zoom factor was set at 1.5 and images were acquired in 120 views with 3 degrees/steps. Reconstruction was done using filtered back projection with Chang’s attenuation correction and a Butterworth filter (0.55 Nyquist frequency and power 10). Images were displayed in the AC-PC plane using a ten-step color scheme in the axial, coronal, and sagittal planes. Detailed lateralizing findings are listed in Table 2. All patients exhibited concurrently: 1) moderately large zones of hypoperfusion, largely in the cortex and subcortical white matter; and 2) smaller zones or foci of hyperperfusion within or adjacent to the regions of hypoperfusion. These alterations were contralateral to the symptoms in 7 patients and ipsilateral in 1 (Case 5). A typical SPECT scan from one of the cases is shown in Fig. 1**,** and Supplemental Figs. 2A–2G show the SPECT scans in the remainder.

**Table 2:** Lateralized SPECT Perfusion Findings*

Location	Patient 1	Patient 2	Patient 3	Patient 4	Patient 5	Patient 6	Patient 7	Patient 8
Frontal	Hypoperfusion	Hypoperfusion	Mixed hyper/hypoperfuison	Hyperperfusion	Hypoperfusion	Mixed hyper/hypoperfuison	Mixed hyper/hypoperfuison	Normal
Parietal	Normal	Hypoperfusion	Mixed hyper/hypoperfuison	Normal	Hyperperfusion **	Normal	Mixed hyper/hypoperfuison	Hyperperfusion
Temporal	Hypoperfusion	Hot spot+ hyperperfusion	Hot spot+hyperperfusion	Normal	Hypoperfuison	Mixed hyper/hypoperfuison	Mixed hyper/hypoperfuison	Hot spot + hypoperfusion
Occipital	Hypoperfusion	Normal	Hypoperfusion	Hot spot + hyperperfusion	Hypoperfusion	Hot spot+hypoperfusion	Hot spot+hypoperfusion	Hypoperfusion
Basal ganglia	Hypoperfusion	Normal	Hypoperfusion	Normal	Normal	Hypoperfusion	Normal	Hypoperfusion
Thalamus	Hypoperfusion	Hypoperfusion	Hypoperfusion	Hypoperfusion	Normal	Hypoperfusion	Hypoperfusion	Hypoperfusion
Cerebellum	Normal	Normal	Normal	Normal	Normal	Hyperperfusion ***	Normal	Normal
Brainstem	Normal	Normal	Normal	Normal	Normal	Normal	Normal	Normal

*All abnormal findings were contralateral to the side of symptoms, except as noted by asterisks

**Hot spot of parietal hyperperfusion in patient 5 was ipsilateral to the symptom

***Cerebellar hyperperfusion in patient 6 was ipsilateral to the symptoms

## Discussion

To our knowledge, this is the first case series of COVID-19 vaccination related focal neurological deficits not due to cerebral venous thrombosis. The great preponderance of patients showed lateralized sensory symptoms and signs, and more than half had unilateral motor symptoms and signs that always occurred ipsilateral to the distribution of sensory symptoms. Delayed headache appeared in half of the patients, and visual symptoms resembling visual aura appeared in one. No patients had aphasia, hemineglect, or other cognitive impairments. The preponderance of patients developed the symptoms within the first 24 h after vaccination. Symptom duration was most commonly for 2–4 days and resolved within 2 weeks in all. The syndrome has a predilection for female and obese patients.

Brain imaging is essential in the diagnosis of ischemic stroke and unusual headaches [3]. The negative MRI and MRA findings in all but one of these patients indicate that the syndrome is not caused by structural brain lesions nor primarily by structural vasculopathies. The absence of diffusion restriction on MRI during the symptoms excludes cerebral infarction as the etiology of the neurological deficits. With the distinct clinical manifestation of transient neurological deficits, the possibility of migraine with aura was raised.

All patients demonstrated consistent findings on SPECT scans obtained while symptomatic of moderately large regions of hypoperfusion mixed with central or adjacent smaller zones of hyperperfusion. These findings were contralateral to the side of clinical presentation in all but one case. Compared with previous reports, most of the SPECT findings in migraine aura represent a hypoperfusion pattern in the acute phase followed by a hyperperfusion pattern [4–9]. A study among patients with familial hemiplegic migraine using arterial spin labeling MRI and SPECT also demonstrated biphasic changes starting from hypoperfusion followed by hyperperfusion during the prolonged aura [10].

A leading potential mechanism for some aspects of this focal clinical syndrome is cortical spreading depression (CSD). The mechanism is thought to be a transient wave of depolarized neurons propagating through the cerebral cortex at a rate of 2–5 mm/min, causing a transient focal neurological deficit [11]. In our case, features consistent with cortical spreading depression include initial positive followed by negative sensory symptoms, the frequent presence of a march of sensory symptoms over minutes, delayed headache in one-half of patients, and moderately large regions of hypoperfusion on SPECT. However, several findings are atypical for classic migraine aura, including symptoms’ duration of days rather than tens of minutes, sensorimotor rather than visual symptom predominance, and prior migraine history in only half of patients. Although a typical migraine aura usually lasts for minutes, prolonged auras lasting more than 1 h have been reported in 12–37% of patients [12]. The International Classification of Headache Disorders (ICHD) classified patients with an aura lasting more than 60 min and less than 7 days as “probable migraine with aura (prolonged aura)”, whereas those who experienced an aura for equal or longer than 7 days are classified as “persistent aura without infarction” [13]. The exact mechanism of this unusually prolonged aura is unknown. It is believed that a reverberating, spreading depression wave caused by the activation of N-methyl-D-aspartate (NMDA) receptors is responsible for the prolongation of these symptoms [14, 15]. In addition, patients with familial hemiplegic migraine due to mutations in membrane channels could have motor auras lasting for several days [16].

The demographic profile of our patients, with a predominance of females of reproductive age (24–48 years), provides some additional support for a migrainous etiology. For the 2 male patients, a BMI of more than 35 was observed, which also provides weak support for a migraine mechanism. A recent population-based study demonstrated that obesity (BMI > 30) was associated with a mildly higher prevalence of migraines, especially for those who are younger than 50, with an odds ratio of 1.66 [17].

Regarding the trigger of this phenomenon, sensory stimulation and psychological stress have been shown to cause migraine exacerbation in vulnerable subjects [18]. Therefore, somatosensory pain or stress related to vaccination could precipitate CSD and migraine processes in susceptible individuals. However, other mechanisms also need to be considered. The presence of small zones of hyperperfusion on SPECT scans may indicate regions of focal inflammation and blood-brain barrier dysregulation triggered by the immune reaction to vaccination. A history of autoimmune disease in 2 patients also provides some support for a potential immune pathogenesis. Consistent with a specific immune mechanism is that the clinical syndrome has so far been observed only in patients receiving CoronaVac and not in patients receiving the ChAdOx1 vaccine, suggesting that the vaccine moiety itself or its adjuvant could potentially be precipitating the response. The prolonged, several-day duration of symptoms could reflect a persisting focal inflammation that triggers recurrent cortical spreading depression. Likewise, patients infected with COVID-19 have reported about 13% co-occurrence of headache. It is hypothesized that cytokines and inflammation may involve multiple organs of tropism, including the brain [19]. In other words, COVID vaccination and COVID-19 may share a common pathway via inflammation. Alternatively, the composition of the vaccines should also be considered as the trigger. As proposed in our previous report, aluminum in the vaccine could disrupt the glutamine–nitric oxide–cGMP pathway, leading to overproduction of nitric oxide and over activation of the NMDA receptors [20, 21]. Currently, no certainty has been confirmed regarding the relationship between these hypotheses and vaccination since no robust pathophysiologic studies have been demonstrated. Further re-evaluation is needed to answer these questions [22]. In our study, animal models could not be done due to several limitations, including restricted vaccine usage. However, the findings in our case series are consistent and should be taken into consideration as one of the potential neurological syndromes following vaccination.

In conclusion, we describe the novel clinical entity of reversible focal neurological syndrome following COVID-19 vaccination. During the attack, functional changes in the brain were demonstrated without evidence of structural abnormalities. The condition was fully reversible in that the patient could return to their daily life within 14 days. Although the clinical features are consistent with migraine with aura, the exact cause remains unknown. We propose that cortical spreading depression related to migraine aura is the contributing mechanism. Further investigations are required to fully understand the pathophysiology of this condition.

## Supplementary Information

**Additional file 1.** Supplement Result 1.

**Additional file 2.** Supplemental Figure 2.

## Data Availability

Data is available with corresponding author upon request.
