# Supplementary material for: Prolonged migraine aura resembling ischemic stroke following CoronaVac vaccination: an extended case series
Source: J Headache Pain. 2022 Jan 21;23(1):13. doi: 10.1186/s10194-022-01385-0 (PMC8777408; doi:10.1186/s10194-022-01385-0)
Supplement: Supplementary file 1 — Additional file 1. Supplement Result 1. [file 10194_2022_1385_MOESM1_ESM.docx]

**Supplement Result 1**

**Case history**

Patient 1 was a 24-year-old woman who was referred to our hospital due to acute left hemiparesis and left hemihypoesthesia after CoronaVac vaccination. Twenty minutes after the injection, she experienced visual disturbance as flashing lights in both eyes, lasting for 5 minutes, followed by severe nausea and vomiting. Twenty minutes later she developed tingling sensation in her left fingers, which progressed to numbness and weakness of the left arm and leg in 15 minutes. Next day, she developed pulsatile headache at left temporal region which radiated to neck, lasting for 2 hours and was relief by ibuprofen. Physical examination revealed grade 3 left hemiparesis and loss of pinprick sensation on the left side. Her past medical history was significant for a perimenstrual migraine without aura. On MRA, a mild irregularity of the periallosal artery was observed and vasospasm cannot be excluded. Therefore, oral nimodipine was given 4 days after the onset and her motor weakness was markedly improved one day after treatment. The sensory symptom remained for the next 9 days and disappeared at day 14.

Patient 2 was a 24-year-old male who developed blurred vision with marked nausea and vomiting 2 hours after CoronaVac vaccination. About 4 hours later, he noticed a tingling sensation in the left arm followed by numbness over left cheek and left side of neck, which progressed to left hand and leg within 1 hour. He also complained of mild weakness of the left hand. One day later, he reported a severe unilateral throbbing headache at the left temporal area radiated to the occipital region, which did not respond to acetaminophen. Physical examination revealed a grade 4 left hemiparesis and decreased pinprick sensation on the left side. Vibration and proprioception senses were within normal limits. Ibuprofen and verapamil were prescribed, and his motor and sensory symptoms markedly improved within 2 days but the headache remained for 2 more days. He has a medical history of occasional unilateral moderate to severe headache 2-3 times per year.

Patient 3 was a 42-year-old woman who developed acute onset of right hemiparesis while walking, which led to right ankle sprain, 7 hours after CoronaVac injection. One day after the initial presentation, during hospitalization, she developed unilateral headache at the right temporal and periorbital region, which responded well after ibuprofen treatment. She also has a history of unilateral headache 2-3 times per year since she was a teenager. Physical examination revealed right pronator drifting along with mild weakness of the right leg. There was no senslory loss Hyperreflexia on the right side was also noted. Nortriptyline and verapamil were prescribed and her symptoms resolved within 3 days.

Patient 4 was a 48-year-old female who presented with numbness of the left arm and weakness of the left side one day after CoronaVac injection. She noticed sensory impairment of the left arm while she was applying steroid cream to her chest and arm for urticarial skin lesion. When she tried to examine herself, she found that she could not feel the sharpness of an object when pressed into her left side of face, left arm, and hand. During the day, she also noticed some weakness of the left arm. She also complained of soreness at the left occipital area radiating to her left shoulder. She waited for 5 days until she felt that the symptoms would not resolve by itself, then came to the hospital. Physical examination revealed mild weakness of left upper extremity with decreased pinprick sensation at left side of face and arm. She was given IV fluid and nortriptyline. Her symptoms resolved in 5 days after observation.

Patient 5 was a 47-year-old man who developed sensory disturbance 2 hours after CoronaVac vaccination. His symptoms started with tingling sensation at his right fingertips which progressed to his right hand more prominent on dorsal aspect within 10 minutes. Two hours later, the same tingling sensation started his right toes and progressed to right foot and ankle. Four hours later, he had numbness at right perioral region, buccal area, and tip of his tongue. The tingling sensation resolved spontaneously overnight but left residual numbness at his right hand and perioral area, which brought him to the hospital on the next day. His past medical history includes hypertension, which was diagnosed a few months ago. Physical examination revealed decreased pinprick sensation at the right hand and right perioral region. There were no weaknesses. His symptoms resolved spontaneously after close observation for 4 days.

Patient 6 was a 29-year-old woman who developed sudden right facial numbness and a few minutes later she felt that the lateral side of her right leg was numb. The leg numbness lasted for only 10 minutes and completely resolved, however, she still reported abnormal sensation over the right corner of her mouth with a mild degree of facial droop. Her symptoms developed at day 7 after the 2^nd^ dose of CoronaVac injection. Physical examination revealed mildly asymmetrical nasolabial folds without other neurological deficits. The abnormal facial sensation and mild facial weakness spontaneously resolved within 4 days during clinical observation.

Patient 7 was a 29-year-old woman who had left hemiparesthesia. She complained of tingling sensation 6 days after CoronaVac vaccination. The tingling sensation started at the left hand and progressed to the arm and left side of her face, perioral region, and leg over 10 minutes. Her past medical history includes endometriosis, which she is currently receiving oral contraceptive pills. Physical examination revealed decreased pinprick sensation at the left side of her face, trunk, arm and leg. Her symptoms spontaneously resolved within 2 days during clinical observation.

Patient 8 was a 40-year-old woman who presented with sensory disturbance after CoronaVac vaccination. Thirty minutes after injection, she developed persistent tingling sensation starting at the left arm and progressed to the left hand. The sensory symptom progressed within 60 minutes. A day later, after woke up, she noticed numbness at perioral area and left cheek. Physical examination revealed decrease pinprick sensation left face, neck, lateral part of left arm and leg. Her symptoms resolved in 12 days after observation.
