# Supplementary material for: Prolonged migraine aura resembling ischemic stroke following CoronaVac vaccination: an extended case series
Source: J Headache Pain. 2022 Jan 21;23(1):13. doi: 10.1186/s10194-022-01385-0 (PMC8777408; doi:10.1186/s10194-022-01385-0)

**Supplemental Figure 2**

**Fig.2 Brain perfusion SPECT of patient 1-7 with arrows pointing to the hot spot**

Fig.2A Patient 1


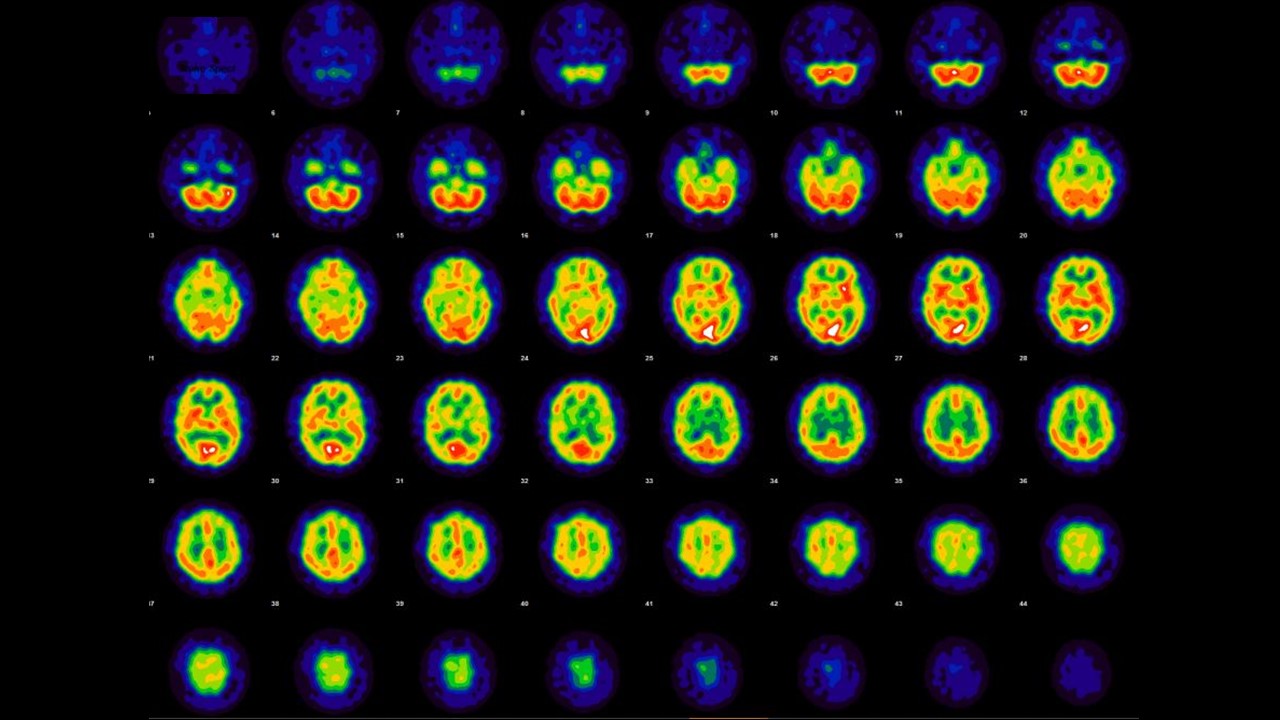


Fig.2B Patient 2


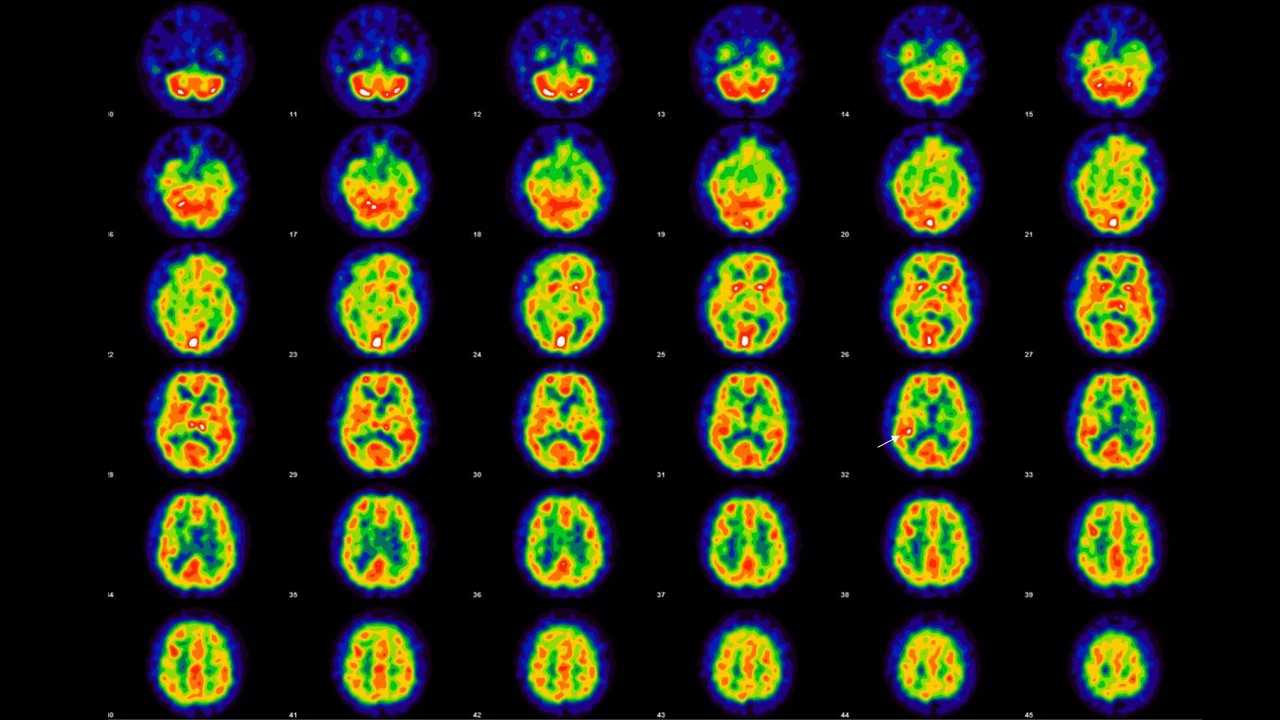


Fig.2C Patient 3


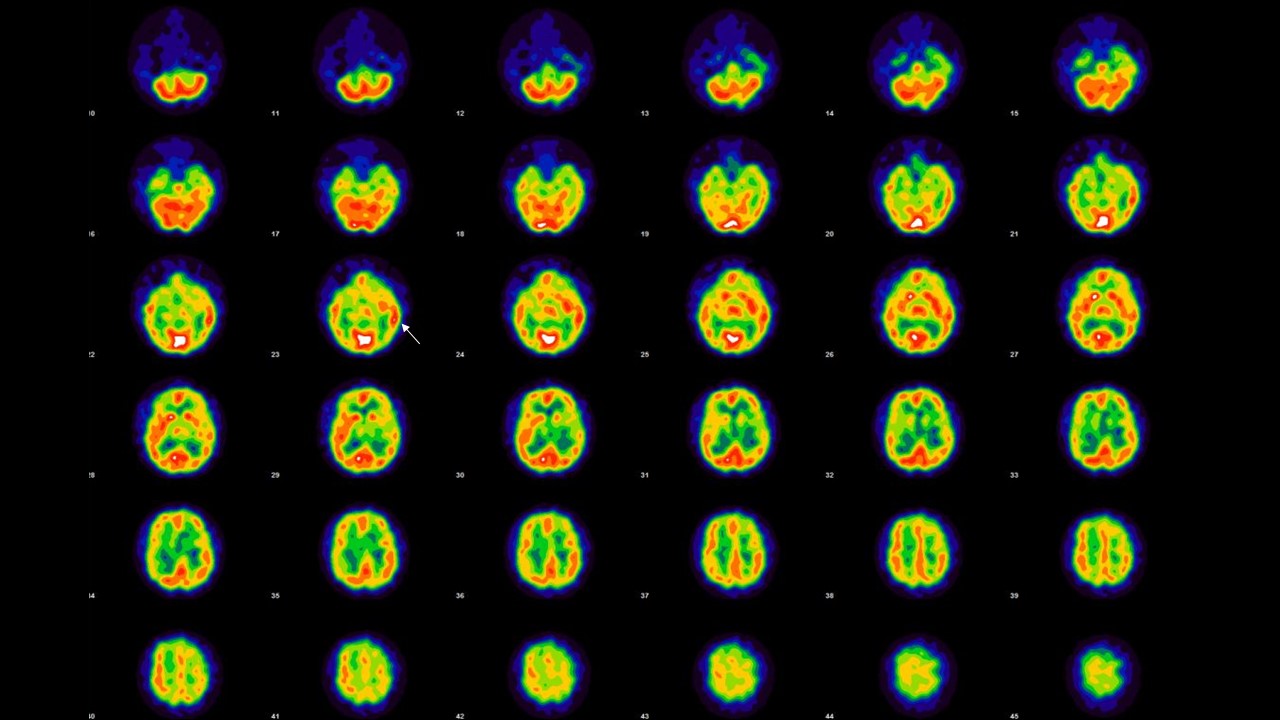


Fig.2D Patient 4


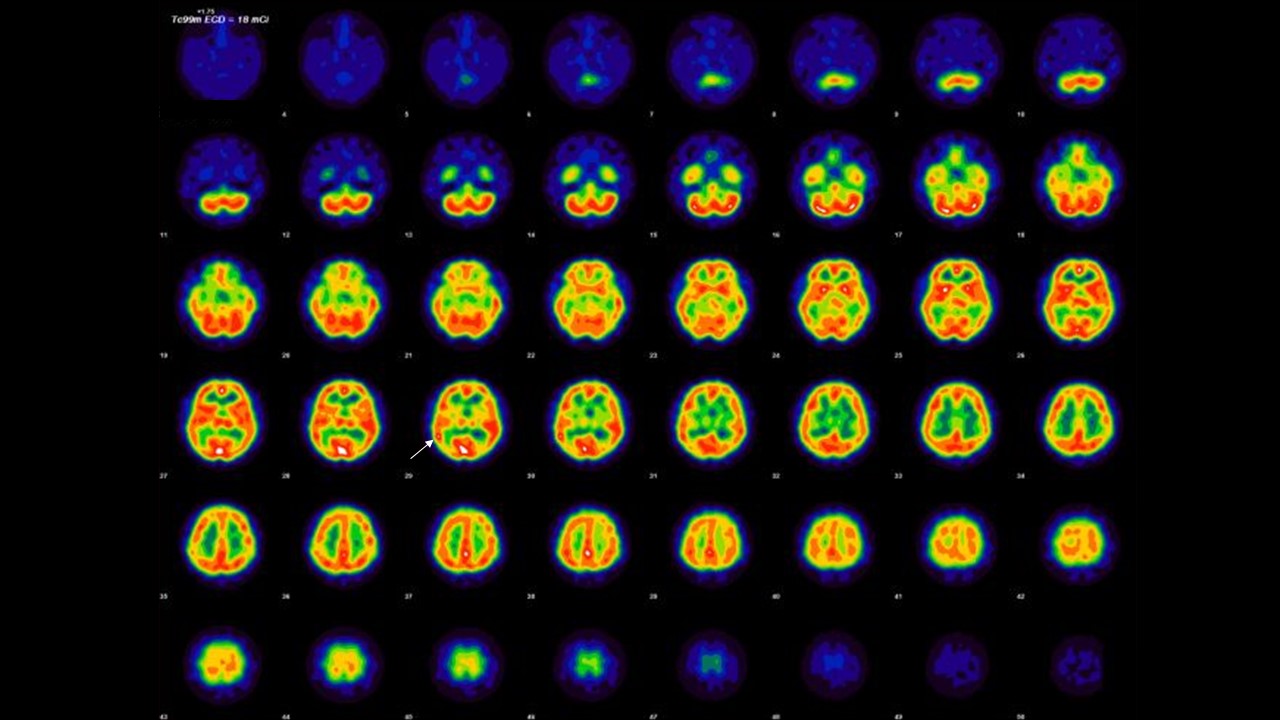


Fig.2E Patient 5


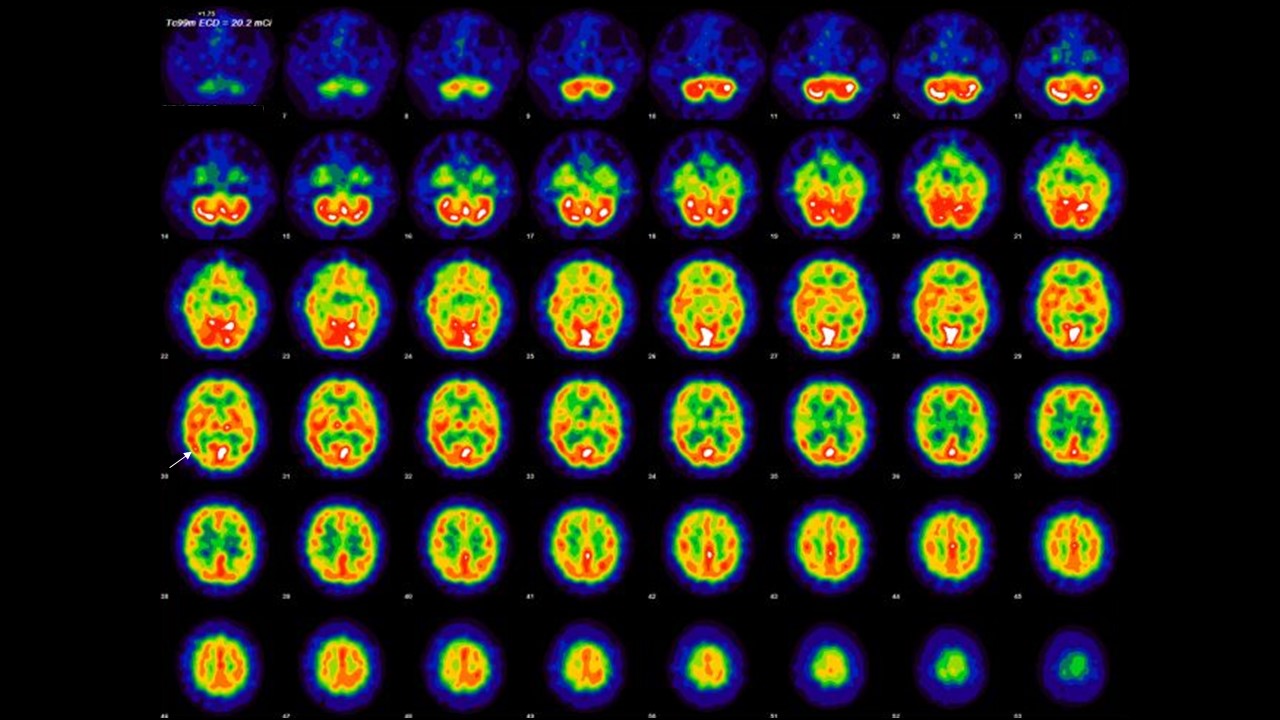


Fig.2F Patient 6


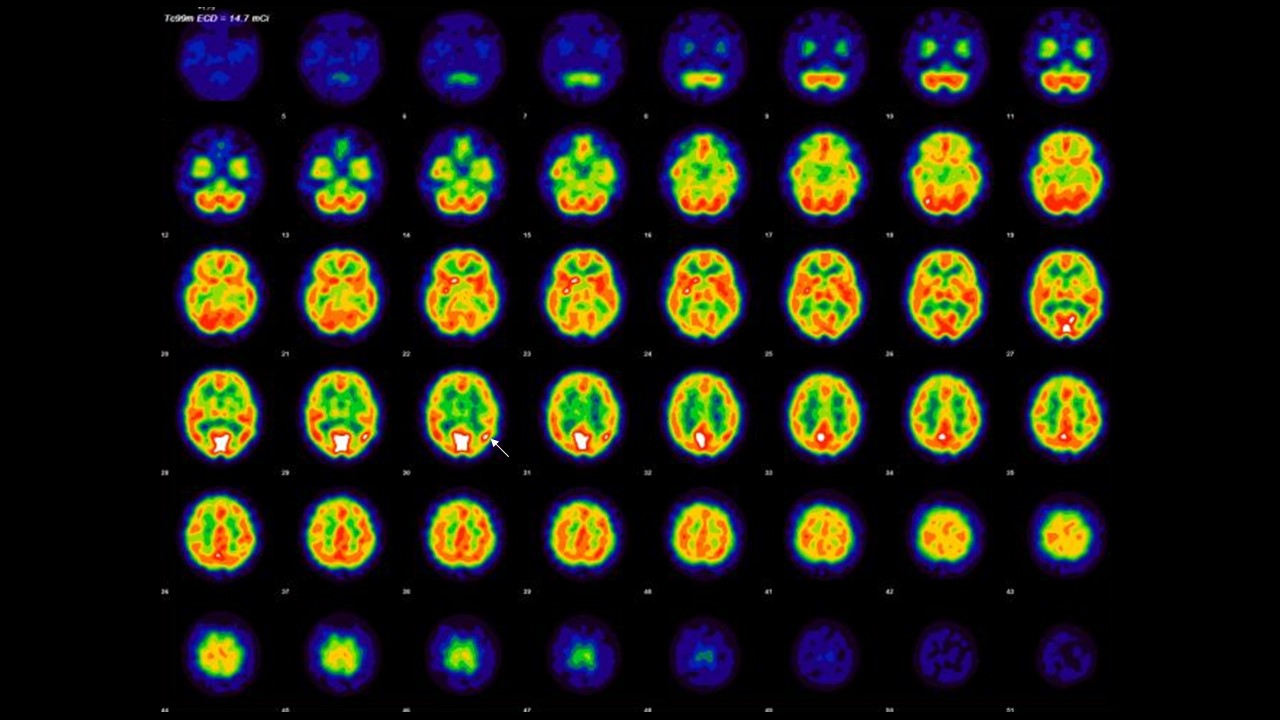


Fig.2G Patient 7


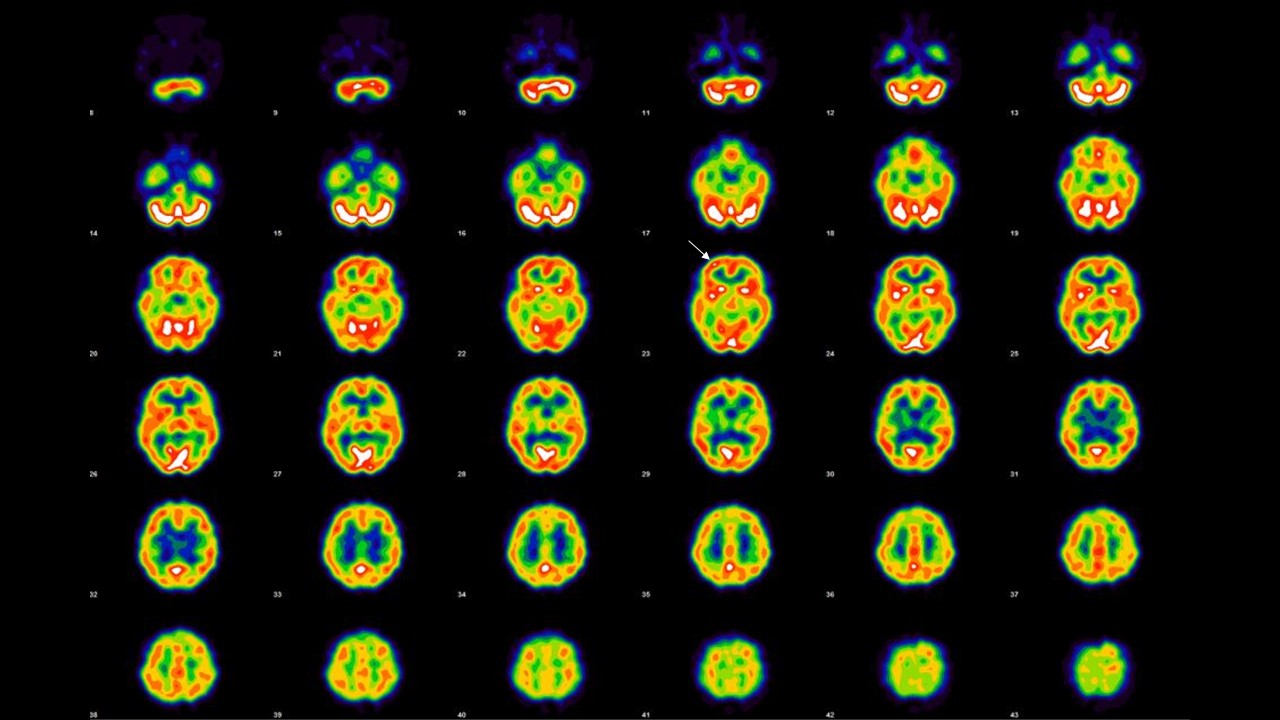

Supplement: Supplementary file 2 — Additional file 2. Supplemental Figure 2. [file 10194_2022_1385_MOESM2_ESM.docx]
